# The hypothalamus as a therapeutic target: Towards novel approaches for managing antipsychotic-induced weight gain

**DOI:** 10.1007/s11154-025-10009-2

**Published:** 2025-12-23

**Authors:** Sayani Mukherjee, Silje Skrede, Johan Fernø

**Affiliations:** 1 Mohn Center for Diabetes Precision Medicine, Department of Clinical Science, University of Bergen, Bergen, Norway; 2 Section of Clinical Pharmacology, Department of Medical Biochemistry and Pharmacology, Haukeland University Hospital, Bergen, Norway; 3 Hormone Laboratory, Department of Medical Biochemistry and Pharmacology, Haukeland University Hospital, Bergen, Norway

**Keywords:** Antipsychotics, Hypothalamus, AMPK, Appetite regulation, Small extracellular vesicles, GLP-1 agonists

## Abstract

Antipsychotic drugs (APDs) represent the cornerstone of pharmacological treatment for psychotic disorders, primarily exerting their therapeutic effects through dopamine D2 receptor antagonism or partial agonism. Their interaction with additional neurotransmitter systems - particularly serotonergic, histaminergic, cholinergic, and adrenergic receptors - has been implicated in the development of metabolic side effects, including weight gain and increased cardiovascular risk. Notably, APDs with the highest therapeutic efficacy, such as clozapine and olanzapine, are also associated with the greatest risk of metabolic disturbances, indicating a complex relationship between symptom improvement and adverse metabolic outcomes. In this review, we explore current evidence on the role of the hypothalamus in APD-induced obesity, with a focus on region-specific neurobiological mechanisms and pathophysiological pathways. The review also evaluates the effectiveness of existing anti-obesity interventions and discusses how these strategies may mitigate metabolic side effects without compromising antipsychotic efficacy. Furthermore, the review presents emerging therapeutic approaches, including clustered regularly interspaced palindromic repeats (CRISPR)/Cas9 gene editing, adenoviral interventions, nano therapies, and small extracellular vesicles targeting hypothalamic function.

## Introduction

Antipsychotic drugs (APDs) constitute the primary pharmacological intervention in the treatment of schizophrenia and other psychotic disorders. Since their introduction in the 1950 s, APDs have evolved and are often categorized into three generations, distinguished by their receptor binding affinity and side effect profiles [1]. First-generation APDs, such as haloperidol and chlorpromazine, are effective in managing positive symptoms like hallucinations and delusions, but are often associated with extrapyramidal side effects, including tremors and involuntary movements like lip smacking [2]. These adverse effects have been reduced in second generation APDs (e.g., clozapine, olanzapine, risperidone, quetiapine, ziprasidone), and even further with the introduction of third-generation agents such as aripiprazole, a partial dopamine agonist [3]. APDs from all three generations are currently utilized in the pharmacological management of psychotic disorders. The selection of a specific agent is primarily driven by clinical judgement, but with emphasis on minimizing adverse effects - especially in the treatment of individuals experiencing a first episode of psychosis [4]. The second-generation APDs, particularly clozapine and olanzapine, have been shown to trigger severe metabolic side effects, such as weight gain, type 2 diabetes, and dyslipidemia, all associated with increased risk for cardiovascular diseases [3]. However, these APDs, with the highest propensity to induce weight gain, appear to have the best therapeutic efficacy, leading to a positive correlation between improvement in symptomatology and the degree of metabolic adverse effects, particularly elevated body mass index (BMI) [3]. Thus, switching from metabolically potent APDs to alternatives with fewer metabolic side effects remains challenging. Consequently, metabolic disturbances continue to pose significant concerns for somatic health and to complicate treatment management in patients with psychotic disorders [5]. Notably, patients who experience weight gain upon APD treatment are almost twice as likely to stop taking APD than those who do not gain weight, associated with significant risk of relapse and suicidality in this patient group [6]. Therefore, it is crucial to explore treatment strategies that could help in mitigating APD-induced metabolic adverse effects while maintaining the effectiveness of the drug. To develop such treatment strategies, it is essential to understand how APDs work in the brain and affect systemic metabolic regulation.

APDs exert their therapeutic effects through the receptor-mediated activation or deactivation of neurotransmitter systems in the central nervous system (CNS). The central mechanism of almost all APDs involves dopamine D2 receptor antagonism or partial agonism [7]. Their propensity to induce metabolic disturbances and weight gain has been associated with affinity to other receptors, including serotonergic (5-hydroxytryptamine; 5HT2C, 5HT1a, 5HT2a, 5HT2c), histaminergic (H1), cholinergic (muscarinic M3, M4), and adrenergic (α and β) receptors [8].

In general, pharmacologically induced weight gain can be caused both by changes in energy intake and expenditure. With regards to APD-induced weight gain, appetite-inducing effects in the hypothalamus that leads to elevated food intake is considered the major driving force, but additional mechanisms are possible [8]. The broad receptor binding profile of APDs makes it difficult to pinpoint the exact contribution of each receptor’s agonism, partial agonism, and antagonism for their effect on energy balance, and the underlying molecular mechanisms are debated [9]. This review evaluates the evidence implicating the hypothalamus in APD-induced obesity, with a focus on region-specific neurobiological mechanisms and proposed pathophysiological pathways. Additionally, we explore the therapeutic potential of hypothalamic modulation as a strategy to attenuate APD-associated weight gain.

## Mechanisms underlying antipsychotic-induced weight gain

### Hypothalamic nuclei involved in the regulation of energy homeostasis

**Fig. 1:**
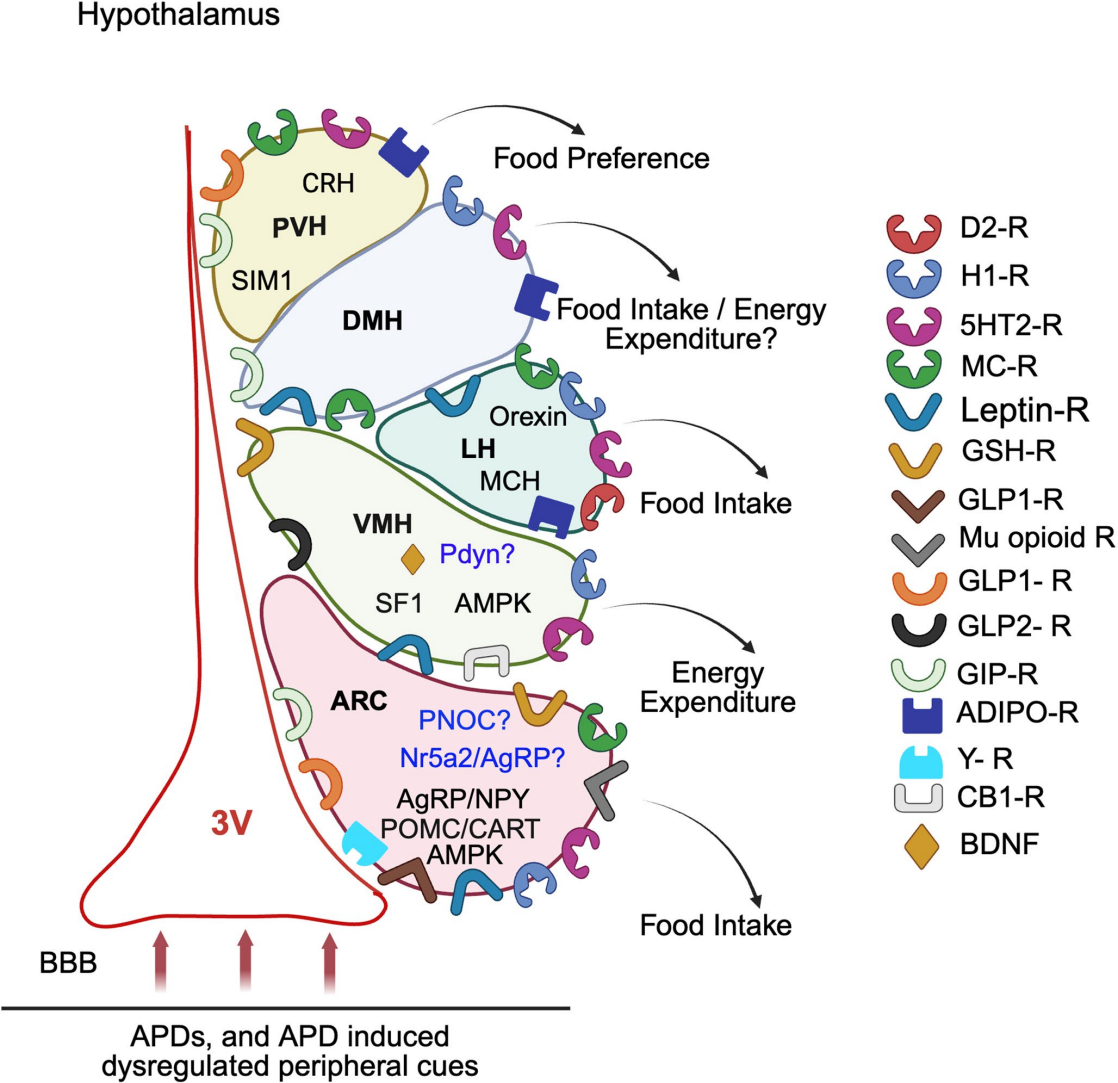
Hypothalamus and APD-induced obesity. This figure illustrates the key hypothalamic nuclei involved in the regulation of energy metabolism and their regulation by APDs and APD-induced dysregulated peripheral cues. APDs and APD-induced peripheral cues cross the blood brain barrier (BBB) to exert their actions in the hypothalamic nuclei such as ARC, VMH, LH, DMH, PVH. Each hypothalamic nucleus contains specific neuronal populations and receptors that regulate food intake, food preference, and energy expenditure. PNOC, Nr5a2/AgRP and Pdyn are highlighted in blue color as emerging target for future research in the context of APD-induced obesity. Abbreviations: Arcuate nucleus (ARC); Ventromedial hypothalamus (VMH); Lateral hypothalamus (LH); Dorsomedial hypothalamus (DMH); Paraventricular hypothalamus (PVH); Corticotropin-releasing hormone (CRH); Single-minded homolog 1 (SIM1) neurons; Steroidogenic factor 1 (SF1); AMP-activated protein kinase (AMPK) neurons, pro-opiomelanocortin/Cocaine- and amphetamine-regulated transcript (POMC/CART); Agouti-related peptide/neuropeptide Y (AgRP/NPY); Prepronociceptin (PNOC); Nuclear receptor subfamily 5 group A member 2/AgRP (Nr5a2/Agrp); Prodynorphin (Pdyn); Dopamine D2 receptor (D2-R); Histamine H1 receptor (H1-R); Serotonin 5HT2 receptor (5HT2-R); Melanocortin receptor (MC-R); Leptin receptor (Leptin-R); Growth hormone secretagogue receptor (GSH-R, also known as the ghrelin receptor); Glucagon-like peptide-1 receptor (GLP1-R); Mu-type opioid receptor (Mu opioid R); Glucagon-like peptide-2 receptor (GLP2-R); Gastric inhibitory polypeptide receptor (GIP-R); Adiponectin receptor (ADIPO-R); Neuropeptide Y receptor (Y-R); Cannabinoid receptor type 1 (CB1-R); Brain-derived neurotrophic factor (BDNF)

Genetic and functional studies in mice and humans have identified key brain circuits regulating appetite and metabolism, with the hypothalamic leptin–melanocortin system as the most well-characterized pathway [10]. This central melanocortin circuit controls energy balance via several key hypothalamic nuclei, such as the arcuate nucleus (ARC), the ventromedial hypothalamus (VMH), the paraventricular hypothalamus (PVH), the dorsomedial hypothalamus (DMH), and the lateral hypothalamus (LH) [10] (Fig. 1). The ARC is primarily known for its role in regulating food intake, containing populations of first-order neurons such as pro-opiomelanocortin (POMC), cocaine- and amphetamine-regulated transcript neurons (CART), agouti–related peptide (AgRP), and neuropeptide-Y (NPY) [11]. These first-order neurons send projections to the other hypothalamic neurons in DMH, VMH, PVH, and LH to control metabolic processes [11]. APDs have been shown to affect the function of the hypothalamus through receptor-mediated increase or decrease in neurotransmitter release [12, 13]. Preclinical studies have revealed that olanzapine acts in the ARC, with antagonistic effects on dopamine (D2)-, H1-, and 5HT2C receptors, alters their mRNA expressions, and promotes food intake by the suppression of POMC neuronal expression while increasing AgRP/NPY expression [14–18]. Supporting a causal role for the 5HT2C receptor in APD-induced weight gain is the reversal of hyperphagia caused by olanzapine and risperidone by co-treatment with lorcaserin, a selective 5HT2C receptor agonist [13, 19]. Moreover, a recent study demonstrated that acute olanzapine treatment increases the expression of mu-opioid receptors (MORs) specifically in the ARC of female rats, and the localization of these receptors on POMC neurons suggests that MOR signaling in the ARC may also contribute to olanzapine-induced hyperphagia [20]. This finding is consistent with a previous study showing that increased MOR signaling in the ARC contributes to hyperphagia and a preference for high fat diet [21]. Clinically, a preference for high fat diet is commonly reported in patients under APD treatment, and this could contribute to gain weight [22, 23]. However, the role MORs in POMC neurons remains unclear. Only about 20% of POMC neurons have been reported to express MORs with a circadian fluctuation in POMC/beta-endorphin levels [24, 25]. MORs are also reported to be present in astrocytes and AgRP/NPY neurons [26–29], suggesting a potential role of these cell types in APD-induced weight gain. Moreover, the reported increase in MOR expression in POMC neurons appears to conflict with the APD-induced reduction in POMC expression described in other articles [18]. Overall, further evidence is needed to understand these mechanisms. Another study showed that olanzapine triggers the transcription factor nuclear receptor subfamily 5 group A member 2 (Nr5a2) that leads to increased expression of AgRP in a subset of neurons, providing more details on how APDs may promote hyperphagia and weight gain in the ARC [30]. AgRP and POMC neurons in the ARC project to melanocortin receptor subtype 4 (MC4R)- expressing neurons in the PVH, where melanocyte-stimulating hormone (MSH) signals to decrease food intake [31]. Preclinical studies have shown that APDs such as olanzapine and risperidone promote hyperphagia and weight gain by inhibiting MC4R-expressing neurons in the PVH [32]. Notably, these effects were reversed by administration of the MC4R agonist setmelanotide, underscoring the role of MC4R in APD-induced weight gain [32]. Similarly, a recent study shows that MC4R inhibition induces a preference for a high-fat diet in mice under gluco-privation state, supporting a mechanism that may parallel APD-induced alteration in food preference [33]. The increased expression of kappa opioid receptor (KOR) and MORs in rat PVH following olanzapine treatment has also been found to be associated with enhanced food intake and body weight gain [34]. DMH may have a role in both food intake and energy expenditure, depending on the neurons that are activated within this area [35, 36]. Olanzapine was shown to disrupt normal leptin-induced activation of DMH neurons, an effect that was significantly reversed by co-administration of the anti-diabetic drug metformin, suggesting that targeting the DMH to alleviate the orexigenic effect of APDs could be a relevant area for further research [37]. The VMH is a hypothalamic nucleus involved in the regulation of thermogenesis, and a recent study demonstrated that prodynorphin (Pdyn)-expressing neurons in the VMH contribute to olanzapine-induced hypothermia and hyperphagia in a mouse model [38]. This effect was mediated through inhibition of VMH-5HT2C receptors and was reversed by chemogenetic activation of the VMH^Pdyn^ neurons. The VMH has also been implicated in the regulation of food intake through mechanisms involving brain-derived neurotrophic factor (BDNF) and MC4R signaling [27]. This is particularly noteworthy because BDNF is a known target of APDs, although primarily in relation to their therapeutic effects rather than their metabolic side effects [39]. Recent findings indicate that BDNF-expressing neurons in the VMH receive input from both AgRP and POMC neurons in the ARC, and that activation of these VMH neurons can counteract the appetite-stimulating effects of AgRP [40]. However, the specific role of the VMH in APD-induced food intake remains unclear and warrants further investigation.

Collectively, these findings highlight the crucial role of the hypothalamic nuclei in APD-induced hyperphagia and weight gain through receptor-mediated mechanisms. Emerging evidence suggests that each of these receptors is linked to intraneuronal signaling cascades that converge on energy sensors like AMP-activated protein kinase (AMPK), either directly or indirectly, which may ultimately mediate APD-induced metabolic dysregulation [41]. In the following section, we will explore how AMPK integrates energy balance signals in the hypothalamic neurons, contributing to APD-induced weight gain.

### Modulation of hypothalamic AMPK by APDs

Hypothalamic AMPK plays a critical role in regulating feeding behavior and energy homeostasis in response to fluctuations in the cellular AMP/ATP ratio [42]. AMPK is activated via phosphorylation, and its functional effects depend on the specific isoforms and spatial distribution of its catalytic subunit -α, and regulatory subunits -β (β1, β2), and γ (γ1, γ2, γ3). The α1 isoform is predominantly expressed in the VMH, whereas the α2 isoform is more broadly distributed in other hypothalamic nuclei [43]. The β1 isoform of AMPK is widely expressed, including in the hypothalamus, while AMPK β2 is more prominent in muscles. Among the γ subunits, γ 2 is the only isoform recently identified in the VMH where it regulates central energy metabolism [44]; γ 1 is ubiquitously distributed, and γ3 is expressed in skeletal muscle. This region-specific activation of hypothalamic AMPK elicits distinct physiological responses, where increased AMPK activation in the ARC promotes food intake, while activation in the VMH suppresses energy expenditure [45, 46]. Both olanzapine and clozapine have been shown to activate hypothalamic AMPK, suggested as a relevant mechanism for APD-induced weight gain and metabolic dysregulation [47–50]. However, findings by Fernø et al. reported that olanzapine could alter orexigenic neuronal signaling without concomitant AMPK activation [16], while a later study from the same group indicated that olanzapine-induced AMPK activation occurs only in the ARC and not the VMH [48]. The activation of AMPK by APDs in the ARC and medio-basal hypothalamus (MBH) was shown to occur through antagonism of histamine H1 receptors and linked to hyperphagia [51, 52]. A review article covering the role of hypothalamic H1 receptors in APD-induced weight gain reports mechanistic differences between short and long term APD treatment. Short term treatments have been shown to activate hypothalamic AMPK-carnitine palmitoyl transferase 1 signaling, whereas in the longer term, brown adipose tissue (BAT) thermogenesis may be reduced (see below) [53]. Notably, the third generation APD-aripiprazole was reported not to alter H1R mRNA expression in the ARC of rats [17], in agreement with its weight neutral profile.

Current evidence has also shown that AMPK in the PVH modulates dietary preference for carbohydrate over fat [54], which could be relevant for APD-induced weight gain. The effects of orexigenic APDs on AMPK activity in the VMH remain less well characterized. APD-induced activation of VMH-AMPK would be expected to promote weight gain by reduced energy expenditure, but emerging evidence suggests that the APD-induced effect on VMH-AMPK is complex and that the effect on peripheral energy expenditure is dose-dependent and influenced by the route of drug administration [55]. More research is needed to evaluate the clinical relevance of these findings. Collectively, the current evidence indicates that APDs do influence AMPK in several hypothalamic nuclei involved in energy regulation, suggesting that the AMPK could be a target for mitigating APD-induced obesity.

## The impact of APDs on peripheral tissues and interplay with hypothalamic regulation of metabolism

Antipsychotic drugs may influence peripheral metabolism through their actions in the hypothalamus. However, they can also exert direct effects on peripheral tissues by altering hormone signaling and modulating key metabolic pathways. In this section, we focus on how APDs impact adipose-derived hormones and directly affect peripheral regulators of lipid metabolism, focusing on AMPK and sterol regulatory element-binding proteins (SREBPs).

### The role of adipose tissue in energy storage and expenditure

APD-induced weight gain is caused by increased white adipose tissue (WAT) mass and leads to changes in the secretion of WAT-derived hormones that may impact metabolic homeostasis. Leptin is an adipokine, i.e. a cytokine secreted by adipose tissue (AT), that acts on the hypothalamic melanocortin system via signaling through the leptin receptor (LEPR) in the MBH [10]. In a physiological setting, leptin levels normally rise with increased fat mass to suppress appetite, while weight loss lowers leptin, and promote food intake [56]. Elevated circulating levels of leptin observed in obesity represent a sign of leptin resistance - a state of impaired leptin signaling that disrupts appetite and energy regulation [56]. In individuals treated with APDs, the association between increased body weight and elevated circulating leptin levels appears to be preserved [57], but circulating leptin levels vary widely among individuals with APD-induced obesity [57–60], suggesting differing degrees of leptin resistance also in this patient group. Interestingly, leptin levels have been observed to rise even before significant weight gain occurs [61]. In rodent models, elevated leptin level has been reported with adipocyte differentiation, and has also been identified as a biomarker for APD-induced weight gain [62, 63], but its predictive value in humans remains uncertain and controversial [64]. The role of leptin in APD-induced obesity and its potential as a biomarker or therapeutic target warrants further investigation.

Like leptin, adiponectin is an essential adipokine with recognized insulin-sensitizing and anti-inflammatory properties. Adiponectin exerts its effects mainly via binding its receptors AdipoR1, and AdipoR2. These receptors are widely expressed in the brain, including the hypothalamus [65]. Adiponectin has been reported to act on hypothalamic ARC-POMC neurons via AdipoR1, leading to increased AMPK activity and increased food intake, but this stimulation is not fully understood and depends on nutritional status [66, 67]. APDs, such as olanzapine and clozapine, have been shown to both increase and decrease serum adiponectin levels and sometimes have a time-dependent (biphasic) effect, which is believed to contribute to metabolic disturbances in humans [68–71]. Consequently, the relationship between circulating adiponectin levels and APD-induced food intake needs further investigation.

In peripheral tissues, such as AT, liver and skeletal muscle, both leptin and adiponectin have been shown to promote fatty acid oxidation and insulin sensitivity, processes where AMPK activation is known to play a major role [72]. AMPK activation suppresses lipogenesis by phosphorylating acetyl-CoA carboxylase 1 (ACC1), a rate-limiting enzyme in de novo fatty acid synthesis, leading to enhanced mitochondrial β-oxidation [72]. Activated AMPK can also inhibit lipogenesis by repressing the cleavage and the subsequent nuclear translocation of SREBPs, particularly SREBP-1c, which normally triggers the expression of fatty acid biosynthesis genes [73]. By inhibiting SREBP activity and lipid biosynthesis, AMPK activation contributes to improved lipid metabolism and insulin sensitivity. Interestingly, numerous in vivo and in vitro studies have shown that APDs can stimulate SREBP-mediated lipogenesis both directly [74, 75] and indirectly, through brain signaling in which hypothalamic JNK activation promotes hepatic Fatty acid synthase (FAS) transcription [76]. This activation of fatty acid- and cholesterol- biosynthesis genes and proteins (via the SREBP1 and SREB2 transcription factors, respectively) has been suggested to be relevant both for the dyslipidemia associated with APDs and for their therapeutic effects [77, 78]. This in accordance with the positive correlation between metabolic disturbances and therapeutic efficacy previously mentioned.

The critical role of BAT in thermogenic adaptation to cold and the maintenance of metabolic homeostasis via activation of the sympathetic nervous system (SNS) in rodents is well established [79]. In the BAT, thermogenesis is mediated by uncoupling protein 1 (UCP1), which triggers mitochondria to produce heat rather than ATP, contributing to energy expenditure [79]. Therefore, the identification of metabolically active BAT in adult humans has generated considerable interest in exploring its potential as a therapeutic target for metabolic diseases [80]. In patients treated with APDs, serum levels of bone morphogenetic protein 8b (BMP8b) - a batokine and marker of mature BAT - were reduced compared to individuals without APD treatment [81]. Preclinically, it has been shown that the thermogenic effect of BMP8b is mediated by the inhibition of VMH-AMPK, with subsequent increase in orexin (OX) signaling in the LH via its receptor OX1R [82]. However, future studies are warranted to evaluate the causal relationship between APD treatment and BMP8b levels in humans. In agreement with the abovementioned stimulation of AMPK by APDs, it was shown in rodents that APDs can reduce sympathetic tone, interfere with UCP-1 action, and impair AT equilibrium, contributing to increased fat accumulation [83–85]. However, other rodent studies have shown that APDs stimulate BAT UCP1 levels and increase energy expenditure despite concomitant weight gain [19, 86]. Furthermore, studies show that BAT activity is also influenced by the dose and the drug administration routes [55, 87].Taken together, these data suggest that the effect of APDs on BAT is complex and that targeting BAT as a treatment strategy to prevent APD-induced weight gain may be challenging. Additionally, the functional significance of human BAT and beige fat remains a subject of debate [88].

## Navigating progress and pitfalls: Therapeutic approaches to APD-induced metabolic adverse effects

Over the decades since antipsychotic-induced weight gain was first recognized as a significant adverse effect, a wide range of interventions targeting dysmetabolic conditions have consequently been explored. Interventions targeting APD-induced weight gain are frequently adapted and evaluated without a comprehensive understanding of the underlying biological pathways. The most commonly used strategies are summarized in the following sections.

### Lifestyle changes

Lifestyle changes, such as diet, physical activity, and alterations in behavioral habits, are the first-line intervention for the treatment of obesity and should also serve as the first step in counteracting APD-induced obesity [89, 90]. Indeed, studies on moderate-high-intensity exercise with dietary interventions in patients during olanzapine treatment yielded significant weight reduction and improved physical condition [91, 92],. Preclinical studies provide insights into mechanisms underlying exercise-induced weight loss in APD-driven obesity and metabolic disorders [93, 94]. In one study, exhaustive exercise was shown to increase peripheral phosphorylation of AMPK as well as serum GLP-1 levels, which in turn improved olanzapine-induced hyperglycemia [95]. Nevertheless, lifestyle changes typically fail to result in long-term weight loss. In the case of APD-induced obesity, these efforts can be even less effective, likely due to additional factors such as negative symptoms of schizophrenia, low motivation and APD-induced sedation, which have been shown to hamper adherence to lifestyle interventions [96]. Given these challenges, novel anti-obesity medications, in combination with lifestyle programs, would be expected to represent a more effective treatment strategy.

### Bariatric surgery

Bariatric surgery directly regulates hypothalamic energy metabolism by modulating the gut-CNS axis, increasing satiety hormone signaling, reducing appetite, increasing insulin sensitivity, and changing the gut microbiota [97]. Although bariatric surgery could serve as an effective strategy for severe obesity and co-morbidities [98, 99], this also comes with some drawbacks. This is a very invasive procedure and is not recommended for all patients with high-BMI obesity. Further, the effectiveness and success rates of bariatric surgery vary among patients, and many patients regain body weight over time. Considering these limitations of bariatric surgery, its long-term success in treating APD-induced morbid obesity remains uncertain. Further, post-surgical metabolic adaptations are important to achieve psychiatric stability among APD-induced patients, suggesting that bariatric surgery is a complicated strategy to combat APD-induced weight gain [100].

### Management through pharmacological agents

#### Metformin

Metformin has been widely used as an anti-diabetic drug since the 1950 s, with a high tolerability and a good safety profile [101]. Metformin may also induce a moderate, but prolonged decrease in body weight [102]. Several studies have investigated whether starting metformin can help prevent weight gain associated with antipsychotic medications. A recent meta-analysis of 12 studies, encompassing 743 patients receiving both metformin and atypical antipsychotics, revealed significant improvements in BMI and insulin resistance [103]. It has been suggested that metformin lowers blood glucose levels by activating AMPK in the muscle and liver [104]. The opposite effect is found in the hypothalamus and in primary cultures of rat hypothalamic neurons, where metformin inhibits AMPK activity and suppress the upregulation of the orexigenic NPY, which is in line with its anorexigenic effect [105]. New evidence highlights additional mechanisms through which metformin may reduce food intake and body weight, including the modulation of microbiota and gut-brain axis [106–109], but targeting the AMPK pathway may still presents a promising strategy for mitigating antipsychotic-induced weight gain and metabolic disturbances [41], (Table 1).

**Table 1:** Approved Pharmacological anti-obesity drugs that May combat APD-induced obesity through hypothalamic metabolic circuit

Drug/Class	Examples	Mode of action in the hypothalamus	Advantages	Disadvantages
Metformin		Inhibits AMPK and NPY gene expression [104, 106].	Widely available, low cost, modest weight loss, well studied in APD-induced weight gain, improves glycemic index weight gain [102].	Associated with many side effects: such as vitamin B12 deficiencies, lactic acidosis, renal problems, GI problems, and hypoglycemia, cognitive issues [135, 169, 170].
GLP 1- agonists	Liraglutide, semaglutide, exenatide	Acts through hypothalamic neuronal circuits [112, 113].	Significant weight loss effects, improves glycemic index [110].	Mostly injectable except oral semaglutide, loss of muscle mass, associated with multiple GI side effects such as nausea, vomiting, loss of appetite, constipation, diarrhea, costly and limited data in APD-induced obesity [8, 119, 171].

#### GLP-1 agonists

GLP-1 receptor agonists such as exenatide (approved in 2005), liraglutide (2010), dulaglutide (2014), and semaglutide (2017) were originally developed for the treatment of type 2 diabetes. Their additional effects on appetite suppression and weight loss have been recognized for some time. However, it was not until 2021 that semaglutide was officially approved as a treatment for obesity [110]. Since then, newer agents have emerged, including tirzepatide—a dual agonist of GLP-1 and GIP receptors (approved in 2022)—and SAR441255, a triple agonist targeting GLP-1, GIP, and glucagon receptors (also approved in 2022). These drugs represent a new generation of highly effective pharmacological treatments for obesity and related metabolic disorders. Mechanistically, GLP-1R agonists act in the CNS, specifically the hypothalamus, to decrease hunger and increase satiety, with additional mechanisms of action mediated via the CNS and peripheral organs [111–113].

The effects of these anti-obesity drugs on patients on APDs is not well-known since this patient group is often left out from clinical trials. However, in emerging studies, GLP-1 receptor agonists have shown potential in mitigating APD-induced metabolic disturbances such as weight gain and insulin resistance [114–117]. A recent multi-center randomized controlled trial found that individuals with schizophrenia, receiving clozapine and treated with semaglutide for 36 weeks, demonstrated a significantly greater weight loss in the intervention group compared to placebo [118]. Semaglutide was found to be safe and well tolerated in this patient population, suggesting that GLP1-agonist serve as a potential strategy to counteract APD-induced weight gain. Indeed, GLP-1R agonists are associated with adverse effects, particularly GI side effects, with potential pharmacodynamic interaction with anti-cholinergic GI adverse effects of APDs [8, 119]. Previous concerns regarding increased risk of suicidality during treatment with GLP-1R agonists, of particular concern in patients with serious mental disorders, were attenuated by recent data [120]. Also, preclinical studies have shown promising effects of GLP-1R agonists on APD-induced metabolic disorders [121–123]. In female rats, while dulaglutide alone modestly reduced food intake without affecting weight gain, the combination of dulaglutide and food restriction led to significant weight loss and improved metabolic parameters [124]. GLP-1R agonists have also been shown to have potential pro-cognitive effects; in a mouse model of diet-induced obesity, liraglutide reduced hypothalamic microglial activation and proinflammatory signaling, suggesting neuroprotection against high-fat diet-induced neuronal damage mediated by blood-brain barrier disruption, oxidative stress, and insulin resistance [125]. Furthermore, a recent study depicts that liraglutide ameliorates clozapine-induced neurotoxicity in mitochondria and improves energy homeostasis in the rodent brain [126]. All these data underscore the potential of GLP-1 receptor agonists to attenuate antipsychotic-induced metabolic side effects, along with preliminary evidence suggesting possible pro-cognitive effects, underscores the need for further clinical studies to evaluate both outcomes - particularly relevant in light of the negative symptoms many patients with psychotic disorders experience (Table 1). However, heterogeneity in GLP-1 receptor agonist action should be considered, as variability exists with reduced responses in patients treated with the most weight -inducing APDs [127]. For example, delayed gastric emptying may influence APD efficacy, suggesting potential pharmacological interactions between APDs and GLP1R agonists [128].There is currently no available data on the effect of tri-agonist in the context of APD-induced weight gain, which could be a subject for future research due to the enhanced effect of these drugs on body weight loss [129]. Similarly, GLP-1/GIP co-agonists like tirzapatide has been shown to modulate hypothalamic neuronal activity and has shown superior weight loss effect [130, 131], but they are expensive, injectable, and understudied in APD-induced obesity. GLP-1/GCGR dual agonists such as cotaglutide modulate hypothalamic neurons and show potential for greater weight loss efficacy, but still under investigation and not yet approved clinically [132].

Notably, a recent preclinical study demonstrated that the GLP-2 analog teduglutide effectively mitigates olanzapine-induced obesity [38]. The study found that teduglutide reduces hyperphagia and weight gain by inhibiting neurons in the VMH through activation of 5HT2C receptors. While clinical trials are needed to confirm its efficacy in humans, these findings suggest that GLP-2 receptor agonism could represent a promising therapeutic strategy for managing antipsychotic-induced obesity.

#### Other pharmacological strategies

Several medications that were initially developed for purposes other than treating obesity have been found to incidentally trigger anti-obesity effects. As a result, they have also been examined for their potential of preventing APD-induced obesity [133, 134]. These include the antidepressants fluoxetine, a serotonin reuptake inhibitor and reboxetine, a NE reuptake inhibitor; orlistat, a pancreatic lipase inhibitor; betahistine, an H1 receptor agonist; topiramate, an anticonvulsant, and finally, a combination of naltrexone, an opioid receptor antagonist, and bupropion, a D2 and NE reuptake inhibitor. However, while the less pronounced dysmetabolic potential of certain agents can be important in the selection of a therapeutic agent for an individual patient, many of these drugs have acquired insufficient success in ameliorating APD-induced weight gain.

In summary, the anti-obesity strategies discussed above - particularly pharmacological interventions - have shown some success in managing APD-induced obesity. Among these, GLP-1 receptor agonists stand out for their therapeutic potential, although the mechanisms underlying their effects in this context remain incompletely understood. However, their use may be limited by suboptimal efficacy, off-target effects, and adverse side effects [135]. Emerging approaches such as gene therapy and nanotechnology-based treatments, while still largely experimental and lacking clinical validation for APD-induced obesity, represent promising new avenues. In the following sections, we will examine both the potential benefits and the challenges associated with these innovative strategies.

## Towards novel treatment strategies for APD-induced weight gain

### Targeted genetic intervention

Recent advances in gene-editing technologies have opened new avenues for potentially targeting hypothalamic circuits implicated in antipsychotic-induced obesity. Two approaches are (i) CRISPR)/Cas9-based genome editing and (ii) adenovirus-mediated gene delivery, which can be used independently or synergistically. CRISPR is being used both for gene discovery and potential therapies, enabling targeted manipulation of genes identified through genome-wide association studies (GWAS) and transcriptomics [136]. This can help uncover how specific genetic variants, such as single nucleotide polymorphisms (SNPs) in hypothalamic neurons or models, influence appetite and energy regulation [137–139]. GWAS studies have highlighted genes like fat mass and obesity associated (FTO), MC4R, LEP, and LEPR as key players in body weight control [140–142]. Notably, obesity-associated variants near FTO and MC4R are located in enhancer elements active in the hypothalamus, validated through CRISPR editing [143, 144]. A detailed spatial map of where these and other genes are located the human hypothalamus (HYPOMAP) was recently published, which enables discoveries of new drug targets, including those for metabolic disorders [145]. While there is notable scarcity of studies specifically addressing genetic factors related to APD-induced weight gain, variants within or near MC4R, LEPR and 5HT2C have been implicated [141, 146]. but these associations await functional verification. While CRISPR/Cas9 is now established for DNA editing in preclinical models, the newly discovered CRISPR/Cas7-11s is an RNA-editing tool that can modify gene expression without permanently altering the genome [147], a key safety concern with traditional CRISPR/Cas9. Its low off-target effects and lack of permanent mutations make it a promising candidate for therapeutic use. Thus, emerging gene-editing technologies are paving the way for functional exploration of genetic associations, unlocking new possibilities for discovery and therapeutic innovation.

Adenovirus-mediated genetic modulation has served as a useful preclinical study tool for gene function rather than a direct treatment option for humans [148, 149]. Adenoviruses, which are non-pathogenic and do not integrate into the host genome, are compatible with long-term exposure [150, 151]. These features have enabled investigation of experimental treatment of obesity and APD-induced obesity in rodent models. As thoroughly discussed, hypothalamic AMPK is a key mediator of energy homeostasis, and constitutive activation of AMPKα1 by intracerebroventricular adenoviruses into the VMH has been reported to control thermogenesis [82, 152]. Similarly, the administration of adenovirus-encoded dominant negative (DN) AMPKα1 isoform in the VMH has been shown to induce thermogenesis and body weight reduction in high-fat diet-fed obese rats and in ovariectomized rats [153]. Adenoviral modulation of hypothalamic AMPK also influences APD-induced obesity. In female rats, DN-AMPK delivery to the ARC - but not the VMH - reduced olanzapine-induced weight gain, suggesting hyperphagia as the primary driver over decreased energy expenditure [48]. Nevertheless, olanzapine has been shown to impact energy expenditure in rodents, but contrary to what would be expected from the weight gain phenotype, olanzapine exposure yielded increased heat production and elevated oxygen consumption [19] as well as UCP1 activation in BAT [86]. Clarifying the clinical relevance of hypothalamic AMPK in APD-induced weight gain and identifying viable therapeutic strategies remains a critical avenue for future research.

### Nanotechnology

Nanotechnology in the medical field has evolved significantly, with improved drug bioavailability, potential to target specific tissues, enhanced drug stability, and controlled drug release. Nano-formulations could aid drug transport across the BBB to exert effects in the brain. Various types of nano-therapies, including nanoparticle (NP) injections, nanoclusters, nanogels, and nanopatches have been explored in preclinical models for treating obesity and metabolic disorders [154], although only a few advancing into clinical trials. Each method provides unique benefits depending on the delivery routes and therapeutic needs. Briefly, NP injections involve direct administration into the body, providing controlled release of the drug [155, 156]. Nano patches are adhesive patches for localized and continuous drug release over time [157]. On the other hand, nanoencapsulation refers to the process of enclosing drugs within nanoparticles to protect them from degradation and release them at a controlled rate Advanced delivery systems such as nanoencapsulation, solid-lipid nanoparticles, and nanogels have improved olanzapine’s efficacy, bioavailability, and metabolic safety in rodents [158–160]. Nanogels enhance uptake and sustained release, while nanoclusters improve drug stability and enable targeted delivery, which has been shown for olanzapine [161]. A recent study in rats has found that co-treatment of gold nanoclusters (AuNCs) with olanzapine ameliorated dysfunctional H1R-AMPK signaling and increased POMC expression in the hypothalamus, leading to reduced hyperphagia and weight gain [162]. Additionally, AuNCs may target other tissues as shown by upregulated expression of thermogenic proteins (UCP1, PGC1-alpha, and PPAR-y) in the BAT as an alternative mechanism that controls energy expenditure and weight gain [162]. All these studies suggest the potential of nano therapy to enhance the therapeutic outcomes of olanzapine, reduce side effects, and improve treatment efficacy. Nevertheless, nano-therapies for APD-induced obesity are still in the early stages, with two main challenges: (a) their efficacy has been tested mostly in preclinical models, and (b) unspecific off-target effects because of NP synthetic chemical composition [163]. The therapeutic effectiveness of nano-therapies could also be compromised by interaction with immune cells like macrophages in the liver and spleen, leading to inflammation and toxicity. These drawbacks highlight the need for safer and more biologically compatible alternatives. This is where naturally derived nanovesicles, such as small extracellular vesicles (sEV), emerge as a potential solution as discussed in the following section.

### SEV-mediated therapy in the APD-induced weight gain

Small extracellular vesicles are nanosized (30–250 nm) lipid outer-layer vesicles released by the cells and involved in inter-cellular and inter-organ delivery of various biomolecules [154, 164, 165]. Recent advances in medical technology have demonstrated that these nanovesicles can serve as effective drug/biomolecule carriers for substances such as DNA, miRNAs, lipids, and proteins [166]. These nanovesicles offer an intriguing alternative to conventional NP-based drug delivery systems due to their unique structure, natural biocompatibility, reduced immunogenicity, and ability to cross the BBB. Unlike synthetic NPs, sEV are naturally derived and can be engineered to carry molecules, facilitating targeted therapy with reduced off-target effects. In mice, it was shown that sEV loaded with AMPKα1-dominant negative constructs under transcriptional control of the VMH-SF1 promoter can enhance thermogenesis and reduce fat deposition without affecting food intake [167]. In this study, the weight loss effect was primarily driven by SNS-mediated UCP1-dependent BAT thermogenesis, resulting in increased energy expenditure. Moreover, another study by the same research group showed that the metabolic benefits and weight reduction induced by peripherally administered SF1-AMPKα1-DN-containing sEV are evident in both diet-induced obesity models and leptin-receptor-deficient db/db mice, suggesting their effectiveness in promoting SNS-driven BAT thermogenesis in various models of obesity [168]. Applying this approach to an APD-induced obesity model could help evaluate the effectiveness of sEV-mediated treatment strategies. However, a key question remains: is targeting the VMH with sEV-AMPK-DN a viable method for modulating APD-induced obesity via BAT thermogenesis? Or would targeting the ARC with AMPK-AgRP-DN be more effective? Using sEV-AMPK-DN to stimulate BAT thermogenesis in olanzapine-induced obese mouse models may introduce complexity and inconsistent data. This is because previous studies suggest that olanzapine itself enhances BAT activity, potentially through similar mechanisms. In contrast, targeting ARC-mediated food intake with sEV-AMPK-AgRP-DN might offer a more straightforward and clinically relevant strategy to counteract APD-induced weight gain. Moreover, while targeting hypothalamic AMPK allows for precision, concerns remain about its long-term impact on brain function, metabolism, and neuronal health. In summary, sEV-based strategies for reducing weight by targeting hypothalamic neurons in APD-induced obesity show promise but require further validation—both at the molecular level and in terms of translational relevance.

## Conclusion

APDs have been used for over 70 years to treat serious psychotic disorders, yet their mechanisms - especially those underlying metabolic side effects - remain poorly understood. Recent research highlights the hypothalamus as a key driver of APD-induced weight gain, with APDs disrupting appetite regulation by altering neurotransmitter and AMPK signaling. Newly identified neuronal populations in the arcuate nucleus (Nr5a2/AgRP; PNOC^ARC^) may also play a role, though their involvement in APD-induced obesity is still unclear. Peripheral AMPK activation and brain-periphery interactions may further contribute to these effects. This review emphasizes the central role of hypothalamic and adipose tissue signaling - particularly AMPK - in APD-induced obesity, and evaluates current and emerging interventions. While lifestyle changes represent first-line treatment, they often fall short in psychiatric populations. Pharmacological options like GLP-1 receptor agonists show promise but have limitations, and bariatric surgery is invasive and suitable for only a few. Emerging strategies such as CRISPR/Cas9, viral vectors, nanotherapies, and sEV offer exciting possibilities, though they remain experimental. Whether targeting a single pathway is sufficient remains uncertain, given the complex, multi-system effects of APDs. Translating preclinical findings to clinical practice is also challenging due to species differences, dosing, and sex-specific responses.

## Data Availability

No datasets were generated or analysed during the current study.
